# Medical Opinion and Movement

**Published:** 1906-08-04

**Authors:** 


### Medical Opinion and Movement.

The Royal College of Physicians of London has appointed the following lecturers for the year 1907: Milroy, Dr. Leonard Rogers, of Calcutta; Gulstonian, Dr. E. Farquhar Buzzard; Lumleian, Dr. G. H. Savage; Oliver-Sharpey, Dr. Halliburton; Croonian, Dr. W. J. R. Simpson; FitzPatrick, Dr. P. H. Pye Smith. The Bradshaw lecture will be delivered next November by Dr. S. J. Sharkey, the FitzPatrick lectures by Dr. Norman Moore, and the Horace-Dobell by Dr. F. W. Andrewes during the same month.

The Norfolk and Norwich Hospital has suffered loss by the retirement of its senior surgeon, Mr. Williams, who was appointed assistant surgeon to the hospital in 1869. Mr. Williams is widely known and respected throughout the county of Norfolk, and has done much to sustain the surgical reputation of the Norfolk and Norwich Hospital. Mr. Williams has been appointed consulting surgeon, and we should hope that he may be able to bring his thirty-seven years' experience to bear upon the management by becoming an active member of the committee.

It is strange how hospital and asylum authorities think the public interests are best served by a policy of covering up and concealing any unusual incidence of infectious disease in public institutions. This is due to a wrong conception of the duties of the medical officer and of other authorities, and tends to create apprehension in the public mind as to whether these officers are not in some way responsible for the outbreak. The public in such circumstances are apt, though usually erroneously, to attribute outbreaks of this type to want of foresight or capacity or to some undefined maladministration. A policy of concealment is apt also to raise the question of adequacy of treatment. For if the authorities do not know what is required, they cannot provide the necessary equipment or the requisite staff, a condition of affairs which has happened more than once of late.

The discussion on the election of direct representatives to the General Medical Council at the annual meeting of the British Medical Association was instructive and interesting. It is hopeful for the future of the medical profession, in the sense that it resulted in absolving all candidates from the pledge contained in the first paragraph of the Association's scheme for the selection of candidates, and that in the end a motion by Mr. Parkinson was adopted, which absolved the British Medical Association from any responsibility in regard to any candidate coming forward at the ensuing election. If the medical profession is ever to occupy its proper position in the opinion of the people of this nation it is essential that the independence and character of individual members shall be maintained. We therefore congratulate the profession that Mr. Parkinson should have induced the annual representative meeting to decide, in view of the difficulties that had arisen in connection with the declarations by candidates, to decline to nominate candidates for election on the General Medical Council.

The work which the National Association for erecting sanatoria for workers suffering from tuberculosis has undertaken must be watched with keen interest by members of the profession. The novelty of the scheme which this association proposes to work is that, the money to defray the cost of erecting a sanatorium having been obtained from donations, the cost of the maintenance of each institution is to be defrayed by the working classes through their trade unions and other organisations. These affiliated friendly and other societies are to contribute towards the maintenance of the institution at the rate of £65 per bed per annum. The scheme involves the after-care of the patients by the institution of a farm colony on a portion of the estate where patients may be partially trained in agricultural and allied pursuits under medical supervision. We presume, though there is no information at present obtainable on the point, that the £65 per bed will include a sum for the remuneration of the medical practitioners under whose care the patients will be placed. The association marks a new departure. Its progress must excite considerable interest, and the scheme itself demands the closest attention of the profession.

At the first general meeting of the British Medical Association on the 24th ultimo the Treasurer showed how the expenses of the Association had increased in 1905 compared with 1901. The increases in the expenses arose under the following headings: salaries, an increase of £1,086; the representative meeting and committees, £1,400; the general association expenses increased by £3,500, spent as to £1,000 on the year-book, and the rest due to increases in legal expenses, printing, postages, reporting, stationery and travelling, and £758 in the editorial department. The Treasurer attributed this great increase in expenditure to the new constitution which came into force in 1902, and created a new department. The railway fares of the representatives and members of committees, with the cost of the new department, amounted to £2,624. Dr. Brassey Brierley stated the position in a nutshell by saying the surplus had come down like an avalanche from £5,000 to £800. The General Secretary explained that the charge for advertisements in the *Journal* had been increased from five guineas to £8 per page, with the result that in the half-year ending June 30 the advertisements have decreased by 554 pages. In spite of this decrease the receipts for the half-year had increased by at least £200, to which should be added a saving of £1,500 for paper, printing, etc. In the result the annual representative meeting appointed a committee of inquiry into the financial position, with the view to such economies of administration as are consistent with maintaining and developing the valuable work now being done for the profession.

